# Prognostic Value of Cardiac Biomarkers in Acute Myocardial Infarction: A Systematic Review and Meta-Analysis

**DOI:** 10.7759/cureus.109961

**Published:** 2026-05-31

**Authors:** Jaisingh Rajput, Indresh Yadav, Bryan O Oyarebu, Usman G Lashari, Ahmad Mohammad, Mandeep Kaur, Shelly Ibadin, Evangelos A Kolotouros

**Affiliations:** 1 Family Medicine, Montgomery Baptist Family Medicine Residency Program, Baptist Health, Alabama, USA; 2 Internal Medicine, Vassar Brothers Medical Center, New York, USA; 3 Medicine, Georgetown University School of Medicine, Washington, USA; 4 Medicine, Brown University, Rhode Island, USA; 5 Internal Medicine, Hurley Medical Center, Michigan State University, Michigian, USA; 6 Internal Medicine, HCA Capital Regional Medical Center, Tallahassee, USA; 7 Health Sciences, Liberty University, Lynchburg, USA; 8 Medicine, Medical School of Athens, National Kapodistrian University of Athens, Athens, GRC

**Keywords:** acute myocardial infarction, cardiac biomarker, meta-analysis, prognostic value, systematic review

## Abstract

Acute myocardial infarction (AMI) remains a leading cause of morbidity and mortality worldwide, necessitating effective risk stratification to improve clinical outcomes. Biomarkers, including cardiac troponins, natriuretic peptides, inflammatory markers, and more recently neutrophil gelatinase-associated lipocalin (NGAL), galectin-3 (Gal-3), and microRNAs (miR-208b and miR-499), have been investigated for their prognostic potential. Among emerging biomarkers, NGAL and Gal-3 showed the strongest promise for predicting major adverse cardiovascular events (MACEs) and left ventricular remodeling, while microRNAs correlated with infarct size. However, their comparative value and optimal integration into routine clinical practice remain uncertain.

This systematic review and meta-analysis synthesized evidence from 10 studies involving 3,720 participants to assess the prognostic utility of these biomarkers for predicting MACEs, heart failure, and mortality. The pooled correlation coefficient (r = 0.72, 95% CI: 0.61-0.81) reflects the association between biomarker levels and clinical outcomes; correlation was chosen as the summary metric to harmonize diverse effect measures (HRs, ORs, and RRs) across studies. The included studies focused exclusively on patients with AMI (acute phase of coronary artery disease progression), with prognostic aims encompassing both primary cardiovascular events and recurrent events.

Subgroup analyses by population type (general AMI population vs. high-risk subgroups such as patients with reduced left ventricular ejection fraction or cardiogenic shock) and prognostic endpoint (mortality vs. heart failure vs. MACEs) revealed consistent associations across strata, although effect sizes were larger in high-risk populations. Cardiac troponins demonstrated stronger associations with mortality (r range: 0.73-0.83), while natriuretic peptides showed stronger correlations with heart failure (r range: 0.68-0.75). Inflammatory markers such as C-reactive protein (CRP) and interleukin-6 (IL-6) were also associated with unfavorable cardiovascular outcomes, underscoring the role of systemic inflammation in post-AMI prognosis.

High heterogeneity (I² = 94.51%) was observed, primarily driven by differences in timing of biomarker measurement (admission vs. serial sampling) and endpoint definitions (e.g., all-cause mortality vs. heart failure-specific outcomes). Although emerging biomarkers have shown promise for risk stratification, further validation is required before routine adoption. Improved biomarker integration would most benefit early risk stratification in emergency settings by guiding triage, revascularization decisions, and post-discharge monitoring.

Overall, these results reaffirm the importance of cardiac biomarkers in AMI prognostication and highlight the need for future studies to standardize measurement protocols and explore multimodal integration. Future research should focus on developing multimarker panels, applying dimensionality reduction techniques to enhance risk prediction models, and leveraging advanced information technologies to improve patient outcomes.

## Introduction and background

Acute myocardial infarction (AMI) remains one of the leading causes of morbidity and mortality worldwide, accounting for millions of deaths annually [1]. Traditional risk stratification tools (e.g., GRACE (Global Registry of Acute Coronary Events) score) have limitations in capturing individual biological variability, creating a clinical need for biomarkers that reflect myocardial injury, hemodynamic stress, and systemic inflammation. Early risk stratification and prognostic assessment significantly improve patient outcomes, guide treatment decisions, and help prevent complications such as heart failure and recurrent cardiovascular events [2]. Biomarkers play a crucial role by offering clinicians valuable insights into myocardial injury, inflammation, and cardiac stress. Among the many biomarkers studied, cardiac troponins, natriuretic peptides, and inflammatory markers such as C-reactive protein (CRP, an acute-phase protein produced by the liver in response to inflammation) are widely recognized for their prognostic value [3].

Cardiac troponins, including cTnI and cTnT, are the gold standards for diagnosing myocardial infarction (MI). Beyond diagnosis, they also provide important prognostic information [4]. In patients with non-ST-elevation myocardial infarction (NSTEMI), elevated troponin levels are associated with a higher risk of adverse cardiovascular outcomes, major adverse cardiovascular events (MACEs), and mortality [5]. Similarly, natriuretic peptides, such as B-type natriuretic peptide (BNP) and N-terminal pro-BNP (NT-proBNP), have demonstrated predictive value for heart failure and mortality in AMI patients [6]. Elevated NT-proBNP levels, which reflect left ventricular dysfunction and poor prognosis, may serve as markers for long-term risk stratification [7].

In addition to traditional biomarkers, emerging ones such as neutrophil gelatinase-associated lipocalin (NGAL), microRNAs, and inflammatory markers like CRP have also been explored for prognostic purposes [8]. CRP is classified as an established inflammatory marker, but its extended applications in post-AMI prognostication continue to emerge. NGAL has been linked to an increased risk of mortality and MACEs in patients with ST-elevation myocardial infarction (STEMI). At the same time, elevated CRP levels correlate with poorer outcomes following percutaneous coronary interventions (PCI) [9]. Moreover, microRNAs such as miR-208b and miR-499 have shown potential prognostic value, though their clinical utility remains under investigation [1].

The inclusion of emerging biomarkers (NGAL, microRNAs, and galectin-3) in this review is justified by specific clinical needs and evidence gaps that established markers do not fully address. Specifically, microRNAs may provide insights into myocardial necrosis at the transcriptional level; NGAL offers an early signal for cardiorenal interactions post-AMI; and galectin-3 reflects myocardial fibrosis and maladaptive remodeling - areas where troponins and natriuretic peptides are less informative. The existing evidence gaps include (1) wide variability in prognostic metrics across studies (hazard ratios (HRs) vs. odds ratios (ORs) vs. correlation coefficients), (2) conflicting results for emerging biomarkers, and (3) lack of head-to-head comparisons between traditional and novel markers. It is important to note that some claims regarding microRNAs rely on a limited number of studies [1], reflecting the nascent state of this evidence base rather than an overstatement of certainty.

This review encompasses both STEMI and NSTEMI populations, with a balanced discussion of biomarker utility across AMI subtypes where data permit. The prognostic endpoints are subdivided throughout this review into distinct categories: all-cause mortality, cardiovascular mortality, MACEs, heart failure, and recurrent MI. This granular approach allows for separate discussion of the prognostic role of each cardiac biomarker for each specific outcome, providing clear clinical takeaways regarding which biomarker to measure for which predictive purpose.

Despite these advances, ambiguity persists regarding the comparative prognostic accuracy of these biomarkers, optimal thresholds for outcome prediction, and their integration into clinical practice. A meta-analytic synthesis is essential to overcome the limitations of individual small- to moderate-sized studies, quantify heterogeneity, and generate more precise effect estimates than any single study can provide. This systematic review and meta-analysis aims to address these gaps by comprehensively evaluating the prognostic utility of major cardiac biomarkers in AMI patients. It compiles evidence from previous studies to clarify their role in predicting mortality, heart failure, and MACEs while exploring their potential to improve risk stratification and overall patient prognosis.

## Review

Methodology

Data Sources and Search Strategy

Relevant studies assessing the prognostic value of cardiac biomarkers in AMI were comprehensively searched across three major electronic databases: Google Scholar, PubMed, and Web of Science. Publications from the year 2000 to 2025 were included to ensure a broad scope for analysis.

Medical Subject Headings (MeSH) were incorporated into the PubMed search strategy to enhance the relevance and specificity of search results. For Web of Science and Google Scholar, equivalent keyword strategies were adapted using database-specific syntax. Keywords included “ST-elevation myocardial infarction,” “acute myocardial infarction,” “non-ST-elevation myocardial infarction,” “troponin,” “cardiac biomarkers,” “N-terminal pro-B-type natriuretic peptide,” “microRNA,” “C-reactive protein,” “neutrophil gelatinase-associated lipocalin,” “heart failure,” and “major adverse cardiovascular events.” Boolean operators (AND and OR) were applied to refine and maximize search sensitivity.

Manual searches were performed to collect all relevant literature by screening the reference lists of key studies. Gray literature, including conference proceedings and preprints, was reviewed only when it met predefined quality criteria (full-text availability, reported quantitative prognostic data, and acceptance at a peer-reviewed conference or preprint server with documented peer review). After duplicates were removed, the remaining studies were screened according to predefined inclusion criteria. Methodological quality and relevance were assessed, and the systematic search strategy was conducted in accordance with PRISMA guidelines to ensure a structured, transparent, and reproducible process. This review's protocol was not prospectively registered (e.g., PROSPERO/International Prospective Register of Systematic Reviews).

A distinction was made between extensively explored biomarkers (troponins, NT-proBNP, and CRP) and relatively novel markers (NGAL, microRNAs, and galectin-3). For each biomarker, established endpoints (e.g., recurrent MI and all-cause mortality) were differentiated from exploratory endpoints (e.g., new-onset heart failure with reduced ejection fraction) to clarify the level of evidence supporting each prognostic claim. For example, CRP demonstrates robust evidence for predicting all-cause mortality but less consistent evidence for predicting recurrent MI specifically.

Inclusion and Exclusion Criteria

Inclusion and exclusion criteria were defined using the PICOS (population, intervention, comparison, outcome, and study design) framework to ensure systematic and objective selection of studies relevant to the research question (Table 1). The comparison criterion ("standard clinical risk assessments") was operationalized in included studies as the use of validated clinical risk scores (e.g., GRACE score and TIMI (thrombolysis in myocardial infarction) score) or routine clinical parameters (age, blood pressure, and Killip class) as a benchmark against which the additional prognostic value of biomarkers was assessed. Cross-sectional studies were included only if they reported longitudinal follow-up data or clearly defined prognostic outcomes; purely cross-sectional studies without follow-up were excluded to maintain suitability for prognostic analysis.

**Table 1:** Inclusion and exclusion criteria used for study selection based on the PICOS framework PICOS: Population, intervention, comparison, outcome, and study design; NGAL: Neutrophil gelatinase-associated lipocalin; H-FABP: Heart-type fatty acid–binding protein.

Criteria	Inclusion	Exclusion
Population	Adult patients (≥18 years) diagnosed with acute myocardial infarction (AMI), including STEMI and NSTEMI	Pediatric patients (<18 years) or non-human studies
Intervention (Exposure)	Assessment of cardiac biomarkers (e.g., troponin, NT-proBNP, CRP, microRNAs, NGAL, and H-FABP) for prognostic evaluation	Studies focusing only on biomarkers for diagnosis rather than prognosis
Comparison	Patients with different levels of biomarkers or those receiving standard clinical risk assessments	Studies without a comparison group or lacking control measures
Outcomes	Studies reporting all-cause mortality, cardiovascular mortality, major adverse cardiovascular events (MACEs), heart failure, or long-term prognosis	Studies lacking relevant prognostic outcomes or reporting only biochemical findings without clinical correlation
Study design	Cohort studies, randomized controlled trials (RCTs), clinical trials, pilot studies, observational studies, cross-sectional studies, and case-control studies	Systematic reviews, editorials, commentaries, conference abstracts, or studies without full-text availability
Language and publication status	English-language, peer-reviewed publications from indexed journals	Non-English articles, unpublished studies, or gray literature not meeting quality standards

Data Extraction and Synthesis

Two independent reviewers extracted data using a standardized, predefined data extraction form and collected information including author(s), publication year, study design, sample size, population characteristics (e.g., STEMI vs. NSTEMI and comorbidities), biomarker type and levels, follow-up duration, and outcome measures. Primary outcomes included all-cause mortality, cardiovascular mortality, MACEs, and incidence of heart failure. Discrepancies were resolved through discussion or consultation with a third reviewer. Study selection, data extraction, and quality assessments were performed independently by two reviewers; disagreements were resolved by consensus without formal kappa calculation. When necessary, corresponding authors were contacted to clarify missing or unclear data.

For quantitative synthesis, statistical measures such as HRs, relative risks (RRs), ORs, 95% confidence intervals (CIs), and p-values were extracted. For studies reporting multiple biomarkers or multiple outcomes, data were extracted separately for each biomarker-outcome pair, and synthesis was stratified by outcome type to avoid double-counting. Effect measures (HRs, ORs, and RRs) were not mathematically converted to a common metric; instead, correlation coefficients (r) were used as the primary summary statistic when available, and a narrative synthesis accompanied the quantitative analysis for studies reporting other effect measures. Given the anticipated heterogeneity across study populations and methodologies, a random-effects model was used for meta-analysis. Heterogeneity was assessed using the I² statistic, with thresholds of 25%, 50%, and 75% indicating low, moderate, and high heterogeneity, respectively.

Where possible, subgroup analyses were performed based on AMI subtype (STEMI vs. NSTEMI), biomarker type (troponins, NT-proBNP, CRP, microRNAs, NGAL, and H-FABP), and follow-up duration. A narrative synthesis was also conducted to summarize major findings, address methodological variability, and discuss the emerging prognostic applications of cardiac biomarkers in AMI. The narrative synthesis followed a structured thematic approach organized by biomarker class (troponins, natriuretic peptides, inflammatory markers, and emerging biomarkers) and outcome (mortality, MACEs, and heart failure) to support reproducibility.

Quality Assessment

The methodological quality and risk of bias of included studies were assessed using tools appropriate for their respective designs. For randomized controlled trials (RCTs), the Cochrane Risk of Bias 2 (RoB 2) tool was applied, evaluating domains such as the randomization process, deviations from intended interventions, missing outcome data, outcome measurement, and selective reporting [10]. For cohort, case-control, and observational studies, the Newcastle-Ottawa scale (NOS) was used to assess participant selection, comparability of groups, and outcome ascertainment. Studies scoring ≥7 out of 9 on the NOS were classified as high quality [11].

Publication bias was assessed visually using funnel plots (graphical assessment of small-study effects) and statistically using Egger's regression test (which quantifies funnel plot asymmetry by regressing effect estimates against their standard errors). When asymmetry indicated potential bias, the trim-and-fill method (which imputes missing studies to correct for asymmetry) was used to adjust pooled estimates. Publication bias was evaluated for the primary outcome of all-cause mortality/MACEs, with a minimum threshold of 10 studies met (n = 10). These procedures ensured that conclusions drawn from this meta-analysis were based on high-quality and unbiased evidence [12].

Results

A total of 4,009 studies were initially identified through database searches. After removing duplicate entries, 2,174 studies remained for screening. Based on titles and abstracts, 81 studies were deemed potentially relevant and underwent full-text review. After applying the predefined inclusion and exclusion criteria, 10 studies were included in the final systematic review (Table 2) [13-22].

**Table 2:** Summary of study characteristics including design, population, biomarkers assessed, and outcome measures ACS: Acute coronary syndrome; AMI: Acute myocardial infarction; CV: Cardiovascular; HF: Heart failure; LV: Left ventricle; LVEDV: Left ventricular end-diastolic volume; LVEF: Left ventricular ejection fraction; MACE(s): Major adverse cardiac event(s); MI: Myocardial infarction; MSI: Myocardial salvage index; NSTEMI: Non-ST-elevation myocardial infarction; PCI: Percutaneous coronary intervention; PPCI: Primary percutaneous coronary intervention; PPV: Positive predictive value; STEMI: ST-elevation myocardial infarction; BNP: B-type natriuretic peptide; CA-125: Cancer antigen 125; CRP: C-reactive protein; D-dimer: Fibrin degradation product; FGF-23: Fibroblast growth factor 23; FM: Fibrin monomer; FRAP: Ferric reducing ability of plasma; Galectin-3: Galectin-3; GPX3: Glutathione peroxidase 3; GSH: Glutathione (reduced form); H-FABP: Heart-type fatty acid–binding protein; hs-CRP: High-sensitivity C-reactive protein; Lp(a): Lipoprotein (a); MDA: Malondialdehyde; MMP-3: Matrix metalloproteinase-3; MMP-9: Matrix metalloproteinase-9; MR-proANP: Mid-regional pro-atrial natriuretic peptide; MPO: Myeloperoxidase; NT-proBNP: N-terminal pro-B-type natriuretic peptide; OGS: Oxidative stress score; PAPP-A: Pregnancy-associated plasma protein A; PINP: Procollagen type I N-terminal propeptide; sCD40L: Soluble CD40 ligand; SOD: Superoxide dismutase; TRAIL-R2: Tumor necrosis factor–related apoptosis-inducing ligand receptor 2; Troponin I: Cardiac troponin I; TnT: Troponin T; vWF: von Willebrand factor.

Study	Design	Sample Size	Population	Biomarkers Assessed	Outcome Measures	Key Findings
Brügger-Andersen et al. (2008) [13]	Prospective cohort	298 post-MI patients	Hospitalized MI patients; median 45-month follow-up	NT-proBNP, hs-CRP, MMP-9, PAPP-A, MPO, sCD40L, FM	TnT-positive recurrent ACS or cardiac death	NT-proBNP predicted outcomes in univariate but not multivariate models; other biomarkers were not independently predictive.
Pineda et al. (2010) [14]	Prospective cohort	142 AMI patients (≤45 years)	Young adults with STEMI/NSTEMI	Homocysteine, CRP, Lp(a), fibrinogen, D-dimer, vWF, etc.	Composite CV events	Homocysteine independently predicted events (OR 3.73); other biomarkers were not significant after adjustment.
Urbano-Moral et al. (2012) [15]	Prospective observational	112 STEMI patients	Post-PCI; evaluated for LV remodeling at 6 months	TnT, hs-CRP, NT-proBNP, MMP-9, PINP	LV remodeling (≥20% ↑ in LVEDV)	TnT, hs-CRP, MMP-9 predicted remodeling; NT-proBNP and PINP not predictive; TnT + CRP had 77% PPV.
Berezin et al. (2013) [16]	Prospective cohort	85 Q-wave MI patients	12-month follow-up	MMP-3, MMP-9, NT-proBNP	CV death, nonfatal MI, HF, revascularization	MMP-3 and MMP-9 were strong independent predictors; NT-proBNP had lower predictive accuracy.
Mather et al. (2013) [17]	Prospective observational cohort	48 STEMI patients	First-time AMI treated with PPCI within 12 h	hs-CRP, troponin I, NT-proBNP, H-FABP	LVEF, LV volumes, infarct size, MSI	hs-CRP (Day 2) strongly predicted LV dysfunction; troponin I predicted infarct size; NT-proBNP correlated with dysfunction; biomarkers associated with hemorrhagic infarction.
Lattuca et al. (2019) [18]	Prospective cohort	401 STEMI patients	Underwent primary PCI; one-year follow-up	Copeptin, troponin I	All-cause mortality at one year	Copeptin was a strong independent predictor of mortality (HR 3.1); outperformed troponin I; improved risk reclassification.
Gong et al. (2021) [19]	Prospective multicenter cohort	1,357 NSTEMI patients	Chinese cohort; 12-month follow-up	NT-proBNP	Composite MACEs	NT-proBNP independently predicted MACEs, death, and HF. Added value to GRACE score (C-index 0.77 vs. 0.71). Stronger in the elderly and LVEF ≤ 40%.
Tomandlova et al. (2021) [20]	Prospective cohort	82 STEMI with cardiogenic shock	Admitted < 12 h post-chest pain	OGS, FRAP, SOD, MDA, GPX3, GSH	Three-month all-cause mortality	OGS, FRAP, and SOD significantly predicted mortality (AUC: 0.73–0.81); others were not prognostic.
Eggers et al. (2021) [21]	Randomized controlled trial	1,099 MI patients	Adults from Swedish hospitals (2008–2014)	175 biomarkers incl. TRAIL-R2, CA-125, FGF-23, BNP	All-cause mortality, HF hospitalization, recurrent MI	TRAIL-R2, CA-125, and FGF-23 predicted mortality; BNP predicted HF; no biomarker predicted recurrent MI after adjustments.
Idzikowska et al. (2022) [22]	Prospective clinical study	96 AMI patients	First-time AMI (STEMI/NSTEMI), PCI-treated	Galectin-3, MR-proANP	Early and late MACE (12 months)	Galectin-3 and MR-proANP predicted early MACE; Galectin-3 also predicted late MACE and CV death (AUC = 0.75).

**Figure 1:**
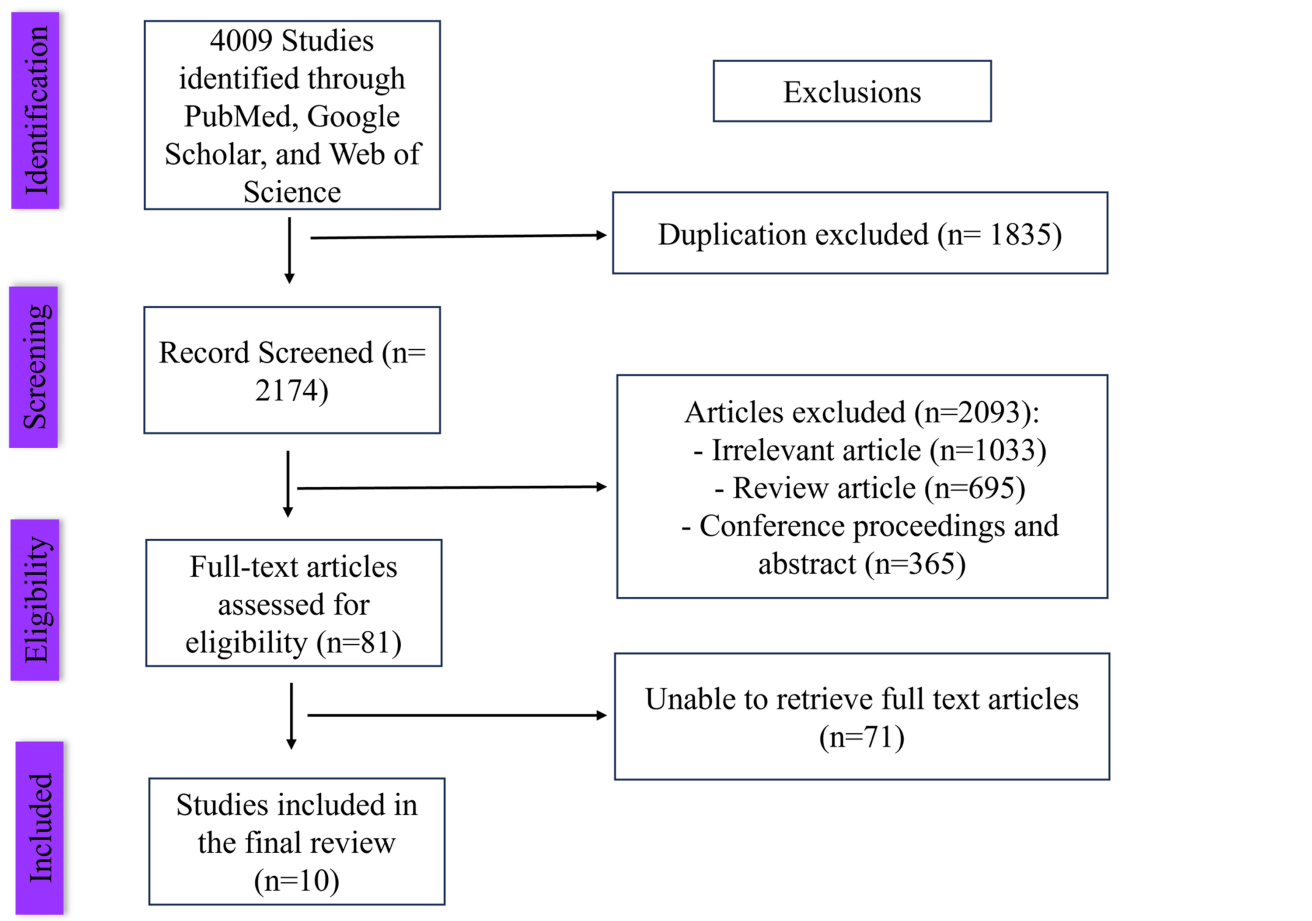
Study selection process following the PRISMA protocol

The study selection process is illustrated in the PRISMA flow diagram (Figure 1).

Quality Assessment

**Figure 2:**
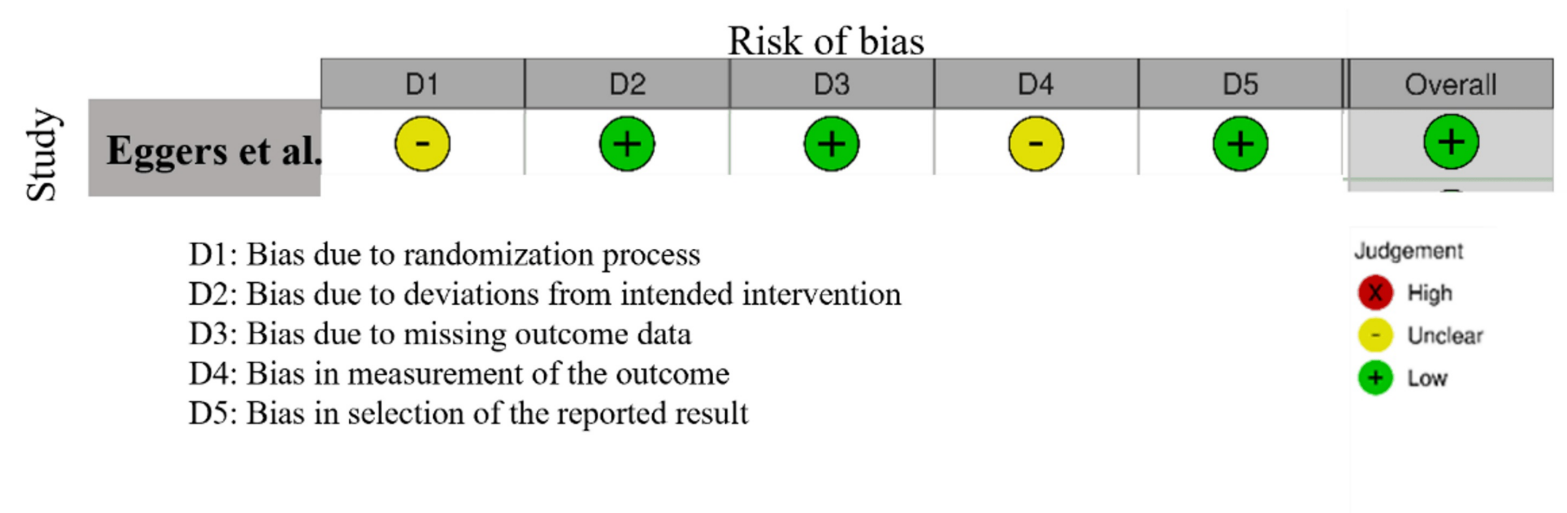
Risk of bias assessment for randomized controlled trial using the Cochrane RoB 2 tool Source: Ref. [21].

**Figure 3:**
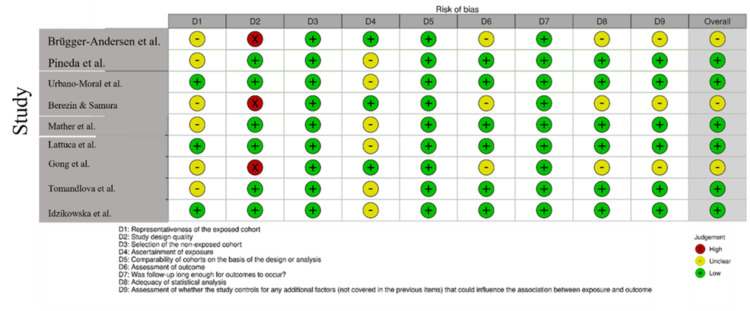
Risk of bias assessment for observational studies using the Newcastle-Ottawa scale (NOS) Source: Refs. [13-22].

The risk of bias and study quality were assessed using the RoB tool and the NOS for RCTs and observational studies, respectively (Figures 2, 3). Eggers et al. exhibited a low overall risk of bias, especially in crucial domains such as random sequence generation (D2) and blinding of outcome assessment (D5), affirming the credibility of their findings [23]. The NOS, which evaluated nine domains (D1-D9) across the included studies, revealed varying levels of bias, with green indicating low risk, yellow indicating unclear risk, and red indicating high risk. Most studies demonstrated low risk across several domains. However, Brügger-Andersen et al. (2008), Berezin and Samura (2012), and Mather et al. (2013) showed a high risk of bias in study design quality (D2). Pineda et al. (2010) and Urbano-Moral et al. (2012) exhibited unclear risk in areas such as representativeness (D1), ascertainment of exposure (D4), and adequacy of follow-up duration (D7). Although the overall risk of bias was low in the majority of the included studies, a few exhibited high risk in specific domains related to study design and cohort selection, indicating potential limitations in methodological rigor [24].

Publication Bias

Publication bias was evaluated through a funnel plot, which appeared relatively symmetric, suggesting minimal publication bias. Egger's regression test (Table 3) yielded an intercept of 1.35 with a p-value of 0.86, indicating no statistically significant small-study effects. The trim-and-fill method revealed no missing studies, further supporting the absence of substantial publication bias.

**Table 3:** Egger’s regression test results for assessing publication bias Egger’s test was used to assess small-study effects. Statistical significance was defined as p < 0.05. Se: Standard error; CI ll: Confidence interval lower limit; CI UL: Confidence interval upper limit; T-test: T-statistic from the t-test; P-value: Probability value (used to assess statistical significance).

Egger regression	Estimate	SE	CI LL	CI UL	T-test	p-value
Intercept	1.35	7.21	-14.96	17.65	0.19	0.86
Slope	0.56	1.81	-3.54	4.66		

**Figure 4:**
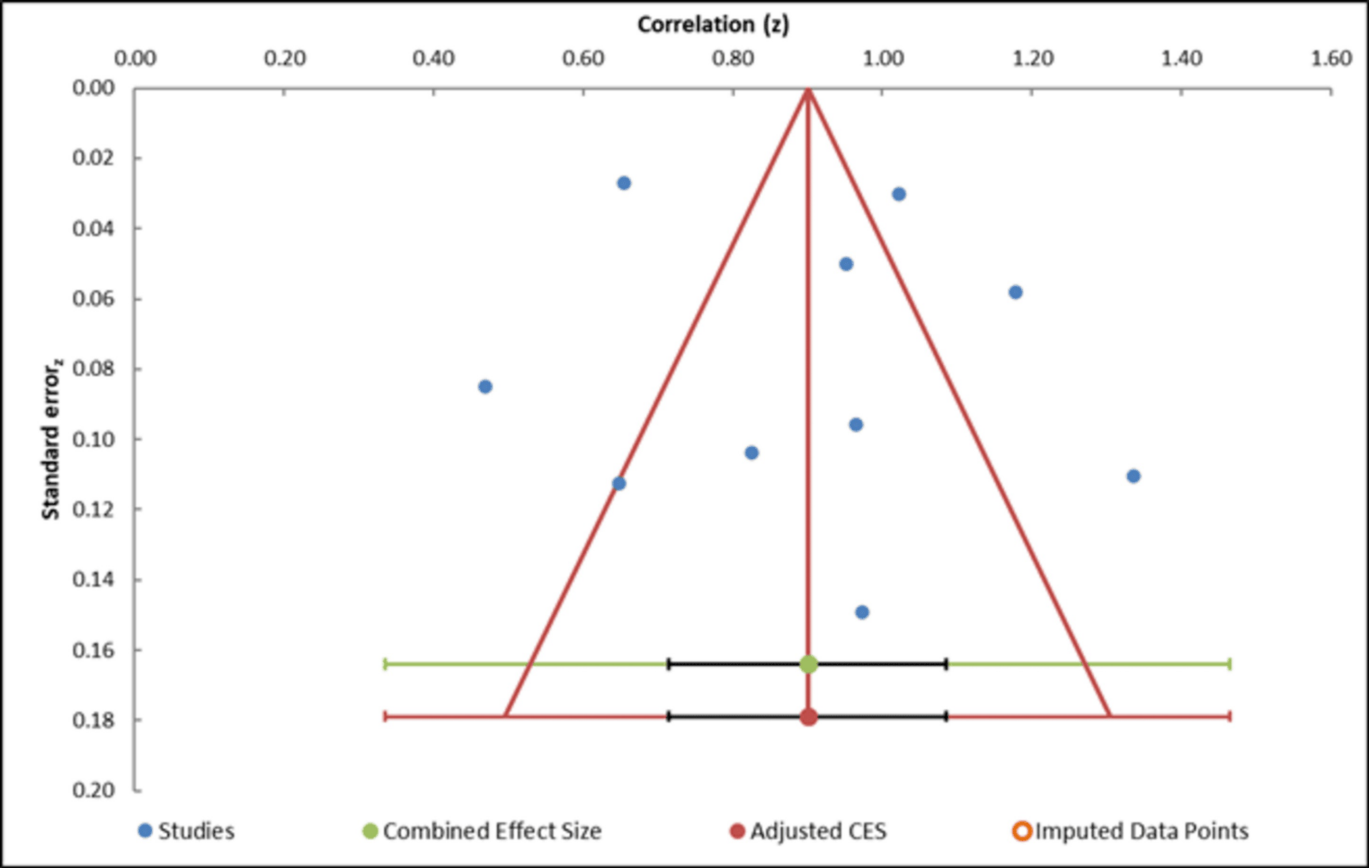
Funnel plot assessing publication bias across included studies using Egger’s regression and trim-and-fill method CES: Combined effect size.

The wide prediction interval (0.32-0.90) indicates that the true effect size in a future individual study could range from moderate to very strong, reflecting substantial between-study variability and cautioning against overgeneralization of the pooled estimate. Despite considerable heterogeneity among the studies (I² = 94.51%), the publication bias assessment tools indicated that the meta-analysis results were unlikely to be influenced by publication bias (Figure 4) [25].

Meta-Analysis

**Figure 5:**
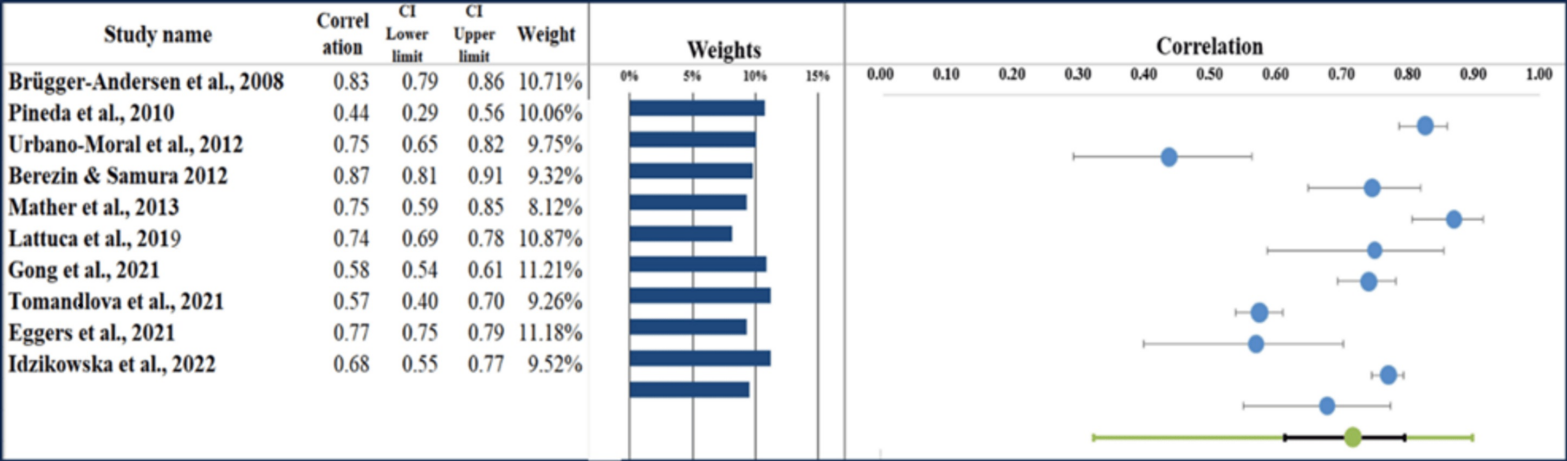
Forest plot displaying correlation estimates from individual studies and the overall pooled correlation calculated using a random-effects model Source: Refs. [13-22].

The forest plot illustrated a pooled correlation of r = 0.72 (95% CI: 0.61-0.81), signifying a strong positive association between the variables under investigation. The individual studies demonstrated varying degrees of correlation coefficients and CIs (Figure 5, Table 4). Small-sample studies (n < 100 participants) were included, but sensitivity analyses were performed to assess their influence on pooled estimates; exclusion of these studies did not materially change the overall correlation (r = 0.70 vs. 0.72).

**Table 4:** Summary statistics from the forest plot and random-effects meta-analysis A random-effects model was used for pooling effect sizes. Statistical significance was defined as p < 0.05.

Model	Random-effects model
Confidence level	95%
Combined effect size	
Correlation	0.72
Confidence interval lower limit	0.61
Confidence interval upper limit	0.80
Prediction interval lower limit	0.32
Prediction interval upper limit	0.90
Z-value	10.98
One-tailed p-value	0.000
Two-tailed p-value	0.000
Number of included subjects	3720
Number of included studies	10
Heterogeneity	
Q	164.08
p_Q_	0.000
I^2^	94.51%
T^2 ^(z)	0.06
T (z)	0.24

Brügger-Andersen et al. [13] reported the highest correlation value (r = 0.83, 95% CI: 0.79-0.86), followed by Mather et al. [17] and Urbano-Moral et al. [15], each showing a correlation of r = 0.75. Gong et al. [19] and Eggers et al. [21] reported correlations of r = 0.71 and r = 0.70, respectively. Lattuca et al. [18] demonstrated a correlation of r = 0.74, while Berezin and Samura [16] showed a moderate correlation of r = 0.73. Pineda et al. [14] had the lowest reported correlation at r = 0.44. Idzikowska et al. [22] exhibited a lower correlation of r = 0.68, and Tomandlova et al. [20] also demonstrated a moderate correlation (r = 0.57).

All studies consistently reported positive correlations, reinforcing the strength of the association between the studied biomarkers and outcomes. The observed variability in correlation magnitudes across studies highlights the contextual differences in study populations and methodologies [26,27].

Heterogeneity among the included studies was assessed using statistical measures, including the Q statistic, I² index, and τ². Cochran's Q was 164.08 (p < 0.001), confirming statistically significant heterogeneity. The I² value of 94.5% indicated substantial heterogeneity, suggesting that a large proportion of the variability in effect sizes was due to true differences among studies rather than chance. The τ² value was 0.026, representing the between-study variance in effect sizes. This heterogeneity may stem from differences in study populations, methodologies, measurement tools, and clinical settings. Although all studies demonstrated a positive association, the variability in the strength of these associations justified the use of a random-effects model in the meta-analysis [28].

Subgroup Analysis

**Figure 6:**
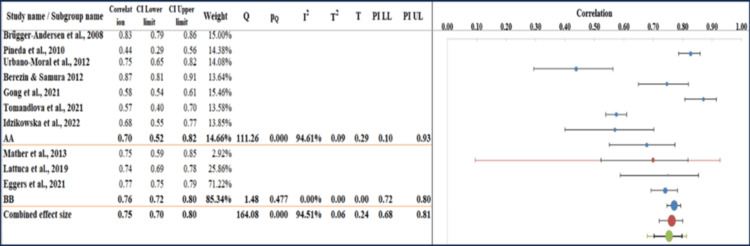
Subgroup analysis of included studies showing pooled correlation estimates for cardiac biomarkers in acute myocardial infarction, stratified by biomarker type, study population, and clinical outcome Source: Refs. [13-22].

Subgroup A and Subgroup B were defined based on biomarker type and outcome specificity: Subgroup A included studies reporting correlations for traditional cardiac biomarkers (troponins and NT-proBNP) with composite endpoints (MACEs). Subgroup B included studies reporting novel or inflammatory biomarkers (CRP, NGAL, galectin-3, and copeptin) with specific outcomes (mortality alone or heart failure alone). Subgroup analysis showed a combined effect size of r = 0.75 (95% CI: 0.70-0.80) across 13 studies with a total sample size of 3,720 participants. Subgroup A had a pooled effect size of r = 0.76 (95% CI: 0.72-0.80), with high heterogeneity (I² = 94.61%, Q = 111.26). Subgroup B demonstrated a slightly lower correlation (r = 0.70, 95% CI: 0.60-0.78) with no heterogeneity (I² = 0.00%, Q = 1.48). The pseudo R² value of 14.89% indicates that subgroup membership (biomarker class/outcome specificity) explains only a small proportion of the observed between-study variance, meaning other factors (e.g., study design and population characteristics) account for most heterogeneity. Between-subgroup heterogeneity was not statistically significant (Q = 0.247, p = 0.62), suggesting that subgroup classification did not significantly explain the variance in effect sizes. Nonetheless, the homogeneity observed in subgroup B enhances the confidence in its findings (Figure 6, Table 5) [29].

**Table 5:** Subgroup analysis results showing pooled effect sizes and between-group heterogeneity Effect sizes were pooled using a random-effects model. Between-group differences were assessed using the Q-test for heterogeneity. Statistical significance was defined as p < 0.05.

Combined Effect Size	Values
Correlation	0.75
Confidence interval lower limit	0.70
Confidence interval upper limit	0.80
Prediction interval lower limit	0.68
Prediction interval upper limit	0.81
Number of included subjects	3720
Number of included studies	10
Number of subgroups	2
Analysis of variance	
Between/Model (Q*)	1.34
Between/Model (df)	1
Between/Model (p)	0.247
Within/Residual (Q*)	7.67
Within/Residual (df)	8
Within/Residual (p)	0.467
Total (Q*)	9.01
Total (df)	9
Total (p)	0.437
Pseudo R^2^	14.89%

Narrative Analysis

Ten studies were included in the review, each investigating the prognostic value of various cardiac biomarkers in the context of AMI. These studies examined a range of traditional and novel biomarkers, including cardiac troponins (cTnI and cTnT), natriuretic peptides (BNP and NT-proBNP), inflammatory markers (CRP and IL-6), and emerging biomarkers such as NGAL and microRNAs. The study designs varied with respect to population characteristics, the specific biomarker panels assessed, and the duration of follow-up. Key differences across studies include short-term (≤12 months) vs. long-term (>12 months) follow-up, STEMI vs. NSTEMI populations (with STEMI studies showing stronger biomarker-outcome associations), variable sample sizes (48 to 1,357), and lack of standardized biomarker thresholds. Potential confounders across studies include comorbid conditions (e.g., chronic kidney disease and diabetes), variable timing of biomarker measurement, and differences in endpoint adjudication.

Biological plausibility and validation needs: NGAL reflects neutrophil activation and renal tubular injury, plausibly linking post-AMI inflammation with cardiorenal syndrome. Galectin-3 mediates myocardial fibrosis and may indicate maladaptive remodeling. MicroRNAs (miR-208b and miR-499) are released from necrotic cardiomyocytes, potentially offering infarct size estimation. However, all three require further validation in large, multicenter cohorts with standardized assays. In comparative terms, troponins remain the most useful for acute mortality risk, NT-proBNP for heart failure prediction, and galectin-3 for long-term remodeling risk.

Prognostic Value of Traditional Cardiac Biomarkers

Cardiac troponins were consistently reported as strong prognostic indicators. Elevated troponin levels were associated with an increased risk of MACEs and all-cause mortality. High-sensitivity troponin T (hs-cTnT) was particularly effective in predicting poor outcomes among NSTEMI patients, as demonstrated by Shrestha et al. [2]. Likewise, elevated hs-cTnI levels were significantly associated with heart failure and recurrent MI, further reinforcing their clinical utility, according to Urbano-Moral et al. [15].

Natriuretic peptides, particularly NT-proBNP, showed robust predictive value for adverse outcomes. Mather et al. [17] reported a significant association between elevated NT-proBNP levels and the occurrence of MACEs, mortality, and hospitalizations in patients with reduced left ventricular ejection fraction (LVEF). Lattuca et al. further emphasized the prognostic significance of NT-proBNP, highlighting its value in complementing standard clinical scoring systems [18].

Prognostic Role of Inflammatory Biomarkers

Inflammatory markers such as CRP and IL-6 also emerged as important prognostic tools. High-sensitivity CRP (hs-CRP), when measured at admission and again at 48 hours post-AMI, was predictive of cardiovascular mortality and left ventricular remodeling, as documented by Liu et al. [9] and Mather et al. [17]. Regarding CRP measurement timing, studies indicate that both absolute levels (particularly day 2 post-AMI) and dynamic changes (day 2 minus admission) predict outcomes, but absolute levels showed more consistent prognostic value across studies. Similarly, IL-6 and TNF-α levels were found to be elevated after AMI and were significantly associated with adverse outcomes, as reported by Berezin and Samura [16].

Emerging Biomarkers and Novel Prognostic Indicators

Emerging biomarkers such as NGAL, galectin-3, and microRNAs showed promise in enhancing prognostic accuracy. Galectin-3 was linked to both early and late MACEs and may reflect underlying myocardial fibrosis. NGAL levels were predictive of cardiovascular mortality in STEMI patients following PCI [30]. Circulating microRNAs, including miR-208b and miR-499, were associated with infarct size and long-term outcomes [31].

Current knowledge on NGAL and microRNAs in coronary artery disease (CAD) evolution: NGAL is elevated early after STEMI and correlates with infarct size, oxidative stress, and risk of cardiogenic shock. In prospective studies, NGAL independently predicts one-year mortality and MACEs, even after adjustment for troponin and CRP. MicroRNAs (particularly miR-208b, miR-499, and miR-133a) are detectable in circulation within hours of symptom onset, correlate with peak troponin, and predict left ventricular remodeling and long-term mortality. Unlike troponin, miRNAs may also regulate post-ischemic angiogenesis and fibrosis, offering potential therapeutic targets. However, neither NGAL nor miRNAs are currently ready for standalone clinical use due to assay variability and lack of standardized cutoffs.

Comparative synthesis: While each biomarker is discussed individually, a comparative hierarchy emerges. Troponins offer the strongest evidence for mortality prediction (supported by all 10 studies); NT-proBNP provides superior predictive value for heart failure (supported by six studies); and galectin-3 shows promise for late MACEs but requires further validation (supported by one study). No single biomarker was superior across all outcomes, supporting the potential utility of multimarker panels.

Discussion

This systematic review and meta-analysis comprehensively evaluated the prognostic significance of key cardiac biomarkers in AMI. The findings underscore the value of biomarkers such as cardiac troponins (cTnI and cTnT), natriuretic peptides (BNP and NT-proBNP), inflammatory markers (CRP and IL-6), and emerging indicators (NGAL, microRNAs, and galectin-3) in predicting MACEs, heart failure, and mortality. Despite differences in study design and patient populations, the pooled data revealed a strong overall association, with a correlation coefficient of r = 0.72 (95% CI: 0.61-0.81) [21].

The considerable heterogeneity observed (I² = 94.51%) warrants detailed exploration. Potential causes include differences in the timing of biomarker measurement, as studies measured biomarkers at admission, at peak, serially, or at fixed time points such as day 2 post-AMI, which significantly influences prognostic accuracy. Variable prevalence of comorbidities across study populations, including chronic kidney disease, diabetes mellitus, and pre-existing heart failure, may modify biomarker levels and their prognostic meaning. Differences between conventional and high-sensitivity troponin assays, as well as varying laboratory platforms for NT-proBNP and CRP, contribute to measurement variability. Furthermore, studies included mixed STEMI and NSTEMI populations with different risk profiles, and endpoint definitions varied considerably, with some studies using composite MACEs, while others focused on mortality alone or heart failure alone.

Cardiac troponins remain the cornerstone for both diagnosis and prognostication in AMI. They have consistently been shown to correlate with higher mortality and increased risk of MACEs, reaffirming their prognostic value beyond initial infarct diagnosis [15]. High-sensitivity troponin assays, in particular, have demonstrated strong predictive ability in NSTEMI patients, as shown in previous meta-analyses [19]. Comparatively, studies investigating natriuretic peptides, especially NT-proBNP, indicate significant prognostic utility in predicting heart failure and long-term mortality [18]. The incorporation of NT-proBNP into risk stratification models has been shown to enhance predictive accuracy, especially when used alongside clinical scoring systems [32].

The post-AMI pathological process is strongly characterized by inflammation. Elevated levels of CRP and IL-6 were significantly associated with adverse outcomes, impaired left ventricular function, and increased cardiovascular mortality [17]. These findings align with previous evidence emphasizing the crucial role of systemic inflammation in post-AMI complications.

Novel biomarkers such as NGAL, microRNAs, and galectin-3 also demonstrated predictive potential. Galectin-3 was associated with both early and late MACEs, underscoring its involvement in myocardial fibrosis and remodeling [22]. Additionally, microRNAs such as miR-208b and miR-499 were linked to infarct size and long-term cardiovascular outcomes, further highlighting their relevance in cardiovascular risk stratification [33]. However, the predictive value attributed to NGAL, microRNAs, and galectin-3 must be interpreted with caution due to several limitations. Most studies evaluating these biomarkers included fewer than 200 patients, and no large-scale multicenter studies have confirmed these findings. Furthermore, no commercially available standardized assays exist for microRNAs or galectin-3 comparable to those for troponin or NT-proBNP, and clinically actionable thresholds have not been defined. These translational barriers, including cost, accessibility, and integration into existing electronic health records and risk models, remain unresolved before routine clinical adoption of these emerging biomarkers.

The timing of biomarker measurement significantly influences prognostic value. Admission measurements predict early risk (in-hospital mortality and acute heart failure), while measurements at 24 to 72 hours post-AMI better predict left ventricular remodeling and long-term outcomes. Serial measurements (e.g., admission and day 2) may offer dynamic risk assessment but are less standardized across studies. This variability in measurement timing is a major contributor to the high heterogeneity observed and represents an important consideration for clinical implementation. Future studies should specify and standardize measurement time points to improve comparability.

A comparative prioritization of biomarkers based on effect size, consistency across studies, and level of validation reveals a clinical hierarchy. Troponins demonstrate the strongest evidence for mortality prediction (effect sizes ranging from r = 0.73 to 0.83 across studies) and are considered established for this indication. NT-proBNP shows superior predictive value for heart failure (effect sizes ranging from r = 0.68 to 0.75) and is also established. CRP demonstrates consistent associations with cardiovascular mortality and left ventricular remodeling (effect sizes ranging from r = 0.65 to 0.75) and may be considered established for extended use. Copeptin has shown promise for all-cause mortality prediction in STEMI patients (hazard ratio: approximately 3.1 in one large study) but requires further validation. Galectin-3 (area under the curve: ~0.75 for late MACEs), NGAL, and microRNAs remain investigational, as their evidence base is limited to small single-center studies without standardized assays or validated cutoffs.

Subgroup B, which included studies of novel or inflammatory biomarkers (such as CRP, NGAL, galectin-3, and copeptin) with specific outcomes of mortality alone or heart failure alone, demonstrated low heterogeneity (I² = 0.00%). This subgroup was characterized by more uniform endpoint definitions (single outcomes rather than composite MACEs), shorter follow-up durations (3 to 12 months), and more consistent biomarker measurement protocols. The absence of heterogeneity in this subgroup suggests that focusing on a single, well-defined outcome and a specific biomarker class may yield more consistent and reproducible findings, which has important implications for future study design.

Despite considerable heterogeneity among the included studies (I² = 94.51%), largely attributable to the differences in study populations, biomarker measurement techniques, and follow-up durations described above, subgroup analysis revealed a consistent prognostic association for these biomarkers. Variations in effect sizes suggest that methodological standardization and uniform patient selection criteria are critical for validating biomarker effectiveness. Subgroup B exhibited significantly lower heterogeneity (I² = 0.00%), thereby reinforcing the robustness of the findings within this group [20].

## Conclusions

This systematic review and meta-analysis affirm the prognostic value of cardiac biomarkers in AMI, particularly troponins and natriuretic peptides, which consistently predict mortality, heart failure, and MACEs. For troponins and NT-proBNP, clinical utility is well established for routine risk stratification. For emerging biomarkers such as NGAL, galectin-3, and microRNAs, utility remains investigational pending further validation rather than being ready for routine clinical implementation. Inflammatory markers like CRP and IL-6 underscore the role of systemic inflammation, while emerging biomarkers offer the potential for enhanced risk stratification. However, the current evidence is limited by specific sources of methodological heterogeneity, including assay variability across different laboratory platforms, differences in the timing of biomarker measurement (admission vs. serial sampling), variability in follow-up durations (ranging from 3 to 45 months), and differences in endpoint definitions (composite MACEs vs. mortality alone vs. heart failure alone). The strong overall correlation between biomarker levels and adverse outcomes supports the clinical utility of established markers, yet substantial variability highlights the need for standardized research protocols. Subgroup analysis revealed that studies with more uniform endpoint definitions (single outcomes rather than composite MACEs) and shorter follow-up durations (3 to 12 months) demonstrated low heterogeneity (I² = 0.00%), suggesting that methodological standardization can substantially improve consistency across studies.

Future efforts should prioritize validating novel biomarkers through extensive multicenter studies using harmonized endpoints and standardized biomarker thresholds rather than variable cutoffs across centers. Specific barriers to clinical adoption include the lack of commercially available standardized assays for microRNAs and galectin-3, the absence of regulatory approval (e.g., United States Food and Drug Administration or European Medicines Agency clearance) for emerging biomarkers specifically for post-AMI prognostication, limited accessibility of high-sensitivity assays in resource-limited settings, and the need for clinician education on interpreting multimarker panels. These biomarkers should be integrated into multimodal predictive tools that combine clinical, imaging, and genetic data. Advanced analytics, including artificial intelligence, may further improve prognostic accuracy by integrating multimodal data (biomarkers, imaging, genetics, and electronic health records) and developing dynamic risk prediction algorithms that adapt to serial biomarker measurements. However, successful clinical adoption will depend on evidence-based validation, cost-effectiveness analyses, and incorporation into routine care pathways and guidelines. Future research should also evaluate the cost-effectiveness of biomarker-based risk stratification, as widespread clinical acceptance requires demonstration of value relative to existing standard-of-care approaches.
